# Informing the consent process for groin hernia repair

**DOI:** 10.1016/j.amsu.2019.02.003

**Published:** 2019-02-20

**Authors:** James Ashcroft, Dania Badran

**Affiliations:** Imperial College London, London, United Kingdom

**Keywords:** Inguinal hernia, Hernia, Herniorrhaphy, Informed consent, Surveys and questionnaires, Patient satisfaction

Following a recent local legal case examining chronic groin pain following hernia repair at West Middlesex University Hospital, the prevalence of chronic groin pain after open inguinal hernia repair within the department was assessed and the departmental surgical consent process was reviewed retrospectively. We read the article ‘Patient's views of the consent process for groin hernia repair: Use of consent template improves compliance with best practice’ by Khan, Bowrey, Williams, Soh, Peleki, Muhibullah, and Waterland with great interest, and share the findings of our department [1].

Questionnaires were posted to 224 patients who underwent an open repair of a single sided inguinal hernia between 1st April 2016 - 1st April 2017 (12 months - 18 month post-operation at time of study). Paper notes, electronic letters, and consent forms were reviewed for 50 patients within the same period. This study defined chronic groin pain as pain persisting longer than 3–6 months [2]. 107 patients responded to the questionnaire. Of respondents, 46% were in pain, discomfort or both (n = 49). 11% were found to be in pain (n = 12) and 6% in both pain and discomfort (n = 6). On review of the consent process, 3 patients were excluded due to lack of information. 64% of patients had evidence of being consented for chronic groin pain (n = 30). Chronic groin pain was most frequently consented by consultants (86%), followed by specialist trainees (PGY-5+) (67%) and senior house officers (PGY-2 to PGY-4) (46%). There was no clear consensus on the ideal time period for consent before a procedure takes place. In this investigation 39/47 patients were consented on day of procedure on ward and in this cohort, 62% had evidence of consent including chronic groin pain. 8/47 patients were consented in clinic between two weeks to six weeks before procedure and in this cohort, 75% had evidence of consent including chronic groin pain.

As highlighted by Khan et al. it is essential for every detail of the consent process to be documented carefully and ultimately the operating surgeon is responsible for consent [1]. This study found alarming results when delegating consent to a responsible clinician with presumed suitable knowledge, suggesting that care must be taken to train those taking consent. Trainees may be unaware of infrequent risks of a procedure which may have great impact on an individual patient's life [1]. Material risks of a proposed surgical intervention must be discussed with great care to the patient and this may require experience beyond that of junior trainees [1].

Patients appear to prefer the consent process to be undertaken in the clinic environment as opposed to on the ward with limited privacy, as Khan et al. have identified [1]. A greater concern found in this study is the impact of factors such as time pressures and distractions on ward based consent, which resulted in a less rigorous documentation of the consent process. The inconsistency in hand written documentation of consent found in the surgical department has stimulated the implementation of information leaflet-consent forms which Khan et al. acknowledge ensure accurate and complete documentation [1].

Informed consent which allows both patient education and empowerment is imperative and therefore it may not be appropriate to apply templates to all patients, as discussed in the recent Montgomery v Lanarkshire case [3]. With the advent of technology within the healthcare system, a safe and comprehensive eConsent process is being introduced within this surgical department and further plans are being made to encourage the use of smart phone applications within consent, to investigate the optimal time and setting for information to be provided to patients before their procedures, and to support the use of patient education groups to enhance the consent process. The department looks forward to utilising the recommendations of Khan et al. to enhance the consent process for groin hernia repair and thanks the group for a thorough and informative study.

## Ethical approval

No ethical approval was necessary for this commentary.

## Sources of funding

No funding was obtained for this commentary.

## Author contribution

JA and DB provided intellectual contributions to this commentary, JA drafted and submitted the response.

## Conflicts of interest

Authors have no conflicts of interest to share.

## Research registration number

No research registration unique identifying number was required for this commentary.

## Guarantor

JA and DB.

## Provenance and peer review

Mot commissioned externally peer reviewed.
